# Consumption of fruits and vegetables associated with other risk behaviors among adolescents in Northeast Brazil

**DOI:** 10.1016/j.rppede.2015.09.004

**Published:** 2016

**Authors:** Fabiana Medeiros de Almeida Silva, Aldemir Smith-Menezes, Maria de Fátima da Silva Duarte

**Affiliations:** a Programa de Pós-Graduação em Educação Física, Universidade Federal de Sergipe (UFS), Aracajú, SE, Brazil; b Instituto Federal de Educação, Ciência e Tecnologia de Sergipe, Universidade Federal de Sergipe (UFS), Aracajú, SE, Brazil; c Centro de Esportes, Programa de Pós-Graduação em Educação Física, Universidade Federal de Santa Catarina (UFSC), Florianópolis, SC, Brazil

**Keywords:** Adolescent, Food consumption, Risk factors

## Abstract

**Objective:**

To determine the prevalence of consumption of fruits and vegetables and identify the association with low level of physical activity, exposure to sedentary behavior, consumption of soft drinks and overweight/obesity in adolescents.

**Methods:**

This is a cross-sectional school-based study with a representative sample of 3992 students aged 14–19 years from the state of Sergipe, Brazil. The outcome was low consumption of fruits and vegetables (<5servings/day). Independent variables were: level of physical activity, sedentary behavior, consumption of soft drinks, and overweight/obesity. Global Student Health Survey questionnaire and body mass and height measurements were used, as well as chi-square test and crude and adjusted binary logistic regression. The significance level adopted was 5%.

**Results:**

The prevalence of inadequate consumption of fruits and vegetables was high – 88.6% (95%CI=87.6–89.5). Higher likelihood of low consumption of fruits and vegetables was verified among boys who were exposed to sedentary behavior (OR=1.63; 95%CI=1.18–2.24), who consumed soft drinks (OR=3.04; 95%CI=2.10–4.40), with insufficiently physical activity (OR=1.98; 95%CI=1.43–2.73) and girls who consumed soft drinks (OR=1.88; 95%CI=1.43–2.47) and those with overweight/obesity (OR=1.63; 95%CI=1.19–2.23).

**Conclusions:**

There is a need of public policies aimed at encouraging the consumption of healthy foods among adolescents.

## Resumo

**Objetivo:**

Determinar a prevalência do consumo de frutas e vegetais e identificar a associação com o baixo nível de atividade física, exposição a comportamentos sedentários, consumo de refrigerantes e sobrepeso/obesidade em adolescentes.

**Métodos:**

Estudo transversal de base escolar com uma amostra representativa de 3.992 alunos entre 14 e 19 anos do Estado de Sergipe, Brasil. O desfecho foi o baixo consumo de frutas e vegetais (<5 porções/dia). As variáveis independentes foram: nível de atividade física, comportamento sedentário, consumo de refrigerantes e sobrepeso/obesidade. Foram usados o questionário Global Student Health Survey e o peso corporal e altura, bem como o teste do qui-quadrado e regressão logística binária (análise ajustada e bruta). O nível de significância adotado foi de 5%.

**Resultados:**

A prevalência de consumo inadequado de frutas e hortaliças foi alta - 88,6% (IC95%=87,6-89,5). Verificou-se maior probabilidade de baixo consumo de frutas e legumes em meninos que estavam expostos a comportamento sedentário (OR=1,63; IC95%=1,18-2,24), que consumiam refrigerantes (OR=3,04; IC95%=2,10-4,40) e com atividade física insuficiente (OR=1,98; IC95%=1,43-2,73) e meninas que consumiam refrigerantes (OR=1,88, IC95%=1,43-2,47) e aquelas com sobrepeso/obesidade (OR=1,63, IC95%=1,19-2,23).

**Conclusões:**

Há a necessidade de políticas públicas destinadas a incentivar o consumo de alimentos saudáveis entre os adolescentes.

## Introduction

In the last decade, encouraging increased consumption of fruits and vegetables has become a public health priority in many countries as a protective effect on the risk of obesity,^1^ type II diabetes mellitus,^2^ cardiovascular diseases^3^ and some types of cancer.^4^ Estimates from the World Health Organization (WHO) show that 2.7 million deaths around the world registered in 2000 could have been avoided with adequate consumption of fruits, legumes and vegetables, reducing by 1.8% the global burden of diseases.^5^

However, in Brazil, this consumption is below that recommended by WHO, which advocates the daily consumption of at least 400g of fruits and vegetables, equivalent to five servings.^6^ On research conducted by VIGITEL telephone survey in Brazilian capitals in 2010, the frequency of adults who consume the recommended amount ranged from 11.3% in Rio Branco (Acre, North Region) to 24.8% in Florianópolis (Santa Catarina, South Region). The lowest frequencies, in males, have occurred in Rio Branco (Acre, 10.0%), Macapá (Amapá, 10.6%) and Aracaju (Sergipe, 11.2%); and, in females, in Rio Branco (Acre, 12.5%), Manaus (Amazonas, 14.9%) and Macapá (Amapá, 15.3%).^7^

Although non-communicable chronic diseases (NCDs) manifest more frequently in adulthood, the identification of health risk behaviors has been widely documented in literature, focusing on young individuals.^8^ Inappropriate eating habits, smoking, alcohol consumption, insufficient levels of physical activity, obesity and excessive time in sedentary leisure activities are some of the most common behaviors during adolescence.^9^ Adolescents show excessive consumption of soft drinks, sugars and snacks like “fast foods” with high fat contents (especially saturated fat) and low intake of fiber and potassium.^10^

Given the above and considering the adequate consumption of fruits and vegetables as a challenge to public health policies in the prevention of diseases, the aim of this study was to determine the prevalence of low consumption of fruits and vegetables among adolescents from public schools in the state Sergipe and its association with low levels of physical activity, exposure to sedentary behavior, consumption of soft drinks and overweight/obesity.

## Method

This study is a secondary analysis of data of an epidemiological cross-sectional survey entitled “Living and Health Conducts of Adolescents Residents in Rural and Urban Areas in the State of Sergipe, Brazil”, developed in 2011 by the Research Group in Physical Education and Health of the Federal Institute of Education, Science and Technology of Sergipe (GPEFiS/IFS). This project was approved by the Ethics Committee on Human Research of the University Hospital of the Federal University of Sergipe (CEP/UFS) under protocol number CAAE - 2006.0.000.107-10.

According to information from the Brazilian Institute of Geography and Statistics,^11^ in 2011, the state of Sergipe, northeastern Brazil, has an area of 21,910.348km^2^, a total population of 2,068,031 people, including 233,119 adolescents (13-19 years). The state has 75 municipalities geographically divided into eight territories.

The study target population was limited to students aged 14-19 years of both sexes, regularly enrolled in the day or night shifts of the State education system. Considering data available on the State Secretariat of Educational (2010), high school enrollment, in 2010, was present for 58,301 students throughout Public State Schools, distributed in 155 teaching units. To compose the research sample, teaching units were randomly selected by the number of students, distributed in municipalities of each territory. Then, classes were randomly selected (considering 25students/class), by grade and shift. The calculation of the research sample used complex sampling process available in the SPSS software (version 15.0). The sample size estimation in the analysis of prevalence for each territory considered the size of the territory population, the estimated prevalence of 50% as the highest expected, confidence interval of 95% and tolerable sampling error of 5%. The sample size was multiplied by 1.5 due to the correction of the design effect (deff=1.5), estimating the need of students for every territory. In order to avoid losing sampling representativeness, an increment of 20% of students were added due reasons such as refusals, age out of the range established in this study, failing to respond to important issues such as sex and age, among others. The association analysis considered also a statistical power of 80% and *Odds Ratio* (OR) of 1.2, requiring a minimum sample of 3875 adolescents for the entire state.

Sample selection used the stratified sampling process by conglomerates in two stages: (1) stratified sampling process proportional to the territory and school size (1=up to 199 students; 2=200-499 students; 3=500 students or more). Thus, considering the three school sizes, it was established as a criterion the random selection of 25% of state education units (155 teaching units), totaling 39 schools distributed in 27 municipalities; (2) classes were selected according to grade and school shift, by using the simple random process considering 25 students per class. The participation of adolescents in the study was voluntary and anonymous, adopting, in addition to the *Parental passive consent form*, the following inclusion criteria: being regularly enrolled in classes from 1st to 3rd grades of selected high schools; being present on the day of instrument application; complete questionnaire response; and age of 14-19 years. School principals also signed the Free and Clarified Consent Form authorizing the participation of institutions in the research.

Data collection was done by physical education teachers trained in the following aspects: domain of the instrument's questions, form of implementation, monitoring, analysis of questionnaires after application and anthropometric measurements. The questionnaire was applied and the anthropometric measurements were taken (body mass and height) at the classroom, with two teachers per class, so that they could answer doubts and assist in filling out questionnaires. The average time applying the questionnaire was 45min. The instrument used was a version of the Global School - based Student Health Survey, proposed by the World Health Organization (GSHS/WHO).

Six health-related behaviors were analyzed: level of regular physical activity, sedentary behavior, and consumption of alcohol, cigarettes, soft drinks, fruits and vegetables. Adolescents who did not practice at least 300min of moderate or vigorous physical activity per week (taking into account physical education classes, displacements, sports practice or other physical activities during leisure time)^12^ were considered insufficiently active. Adolescents exposed to screen (television and media) for more than two hours per day were considered as exposed to sedentary behavior;^13^ consumption of cigarettes and alcohol was considered for those who reported any consumption in the last 30 days; consumption of soft drinks was considered if consumption was reported once or more times per day; overweight/obesity was defined by a body mass index (BMI) - for-age^14^ of >25kg/m^2^. For the consumption of fruits and vegetables, the dependent variable of this study, two or more servings of fruits and three or more servings of vegetables per day were considered as adequate.^6^ In the case of fruits, only the consumption of fresh fruits was considered, in other words, fruit juices were not calculated.

The Intelligent Character Recognition-Teleform software (HS Informática, Rio de Janeiro, Brazil) and the FI-6230 scanner (Fujitsu, Tokyo, Japan) were used to accomplish the tabulation of data, using optical reader. After this phase, the authors manually checked and corrected errors on questionnaires that showed problems.

Data analysis was performed using the Statistical Package for Social Science for Windows software (SPSS, version 20.0). Variables were analyzed using descriptive and inferential procedures. In the bivariate association analysis, the chi-square test for heterogeneity and for linear trend was used. In the multivariable analysis, crude binary logistic regression for the study outcome was used (consumption of fruits and vegetables), being adjusted for age and skin color. The significance level adopted was *p*≤5%.

## Results

Data of 4717 students were analyzed; however, 725 were excluded for the lack of important variables such as age and sex, and also those aged over 19 years. The final sample comprised 3992 children (61.3% female and 38.7% male), aged 14-19 years (51.8% with 16-17 years old). Table 1 shows the demographic characteristics and socioeconomic conditions of individuals participating in the study.

**Table 1:** Sociodemographic characteristics of the students (n=3992) according to sex.

	Female			Male		*p-value*
	N (1544)	%		N (2448)	%	
*Age group*						
14-15	259	16.8		470	19.2	<0.001
16-17	759	49.2		1310	53.5	
18-19	526	34.1		668	27.3	

*Situation of the domicile*						
Urban areas	982	63.6		1484	60.6	0.032
Rural areas	562	36.4		964	39.4	

*Family head*						
Mother	542	35.1		964	39.4	<0.001
Father	879	56.9		1217	49.7	
Others	123	8.0		267	10.9	

*Mothers education level*						
With no education	162	10.5		294	12.0	<0.001
ES not completed	738	47.8		1281	52.3	
ES completed	175	11.3		228	9.3	
HS completed	234	15.2		367	15.0	
College completed	141	9.1		154	6.3	
Unknown	94	6.1		124	5.1	

*Adolescent grade*						
1st year	679	44.0		971	39.7	<0.001
2nd year	502	32.5		841	34.4	
3rd year	363	23.5		636	26.0	

*School shift*						
Day shift	956	61.9		1697	69.3	<0.001
Night shift	588	38.1		751	30.7	

*Family income*						
Up to 1 MS	416	26.9		867	35.4	<0.001
From 1 to 2 MS	589	38.1		965	39.4	
Above 2 MS	511	33.1		557	22.8	
Unknown	28	1.8		59	2.4	

ES, elementary school; HS, high school; MS, minimum salary.

Most students reported living in urban areas (61.8%). For 52.5% the father was the family head, in 50.6% mother had incomplete elementary school, 41.3% of the students were enrolled in the first year of high school, and 66.5% in the day shift (66.5%). Most students had family incomes up to two minimum wages.

In relation to health risk behaviors shown in Table 2, the following prevalence's were found: (a) students with insufficient levels of physical activity − 77.5% (95%CI=76.2-78.8); (b) students exposed to sedentary behavior − 46.7% (95%CI=45.2-48.3); (c) students that did not meet recommendations for the consumption of fruits and vegetables − 88.6% (95%CI=87.6-89.5); (d) students with excessive soft drinks intake − 57.5% (95%CI=56.0-59.0); (e) students with overweight/obesity − 15.5% (95%CI=14.4-16.7); (f) students who reported alcohol intake in the last 30 days − 49.2% (95%CI=47.7-50.8); and (g) students who reported smoking in the last 30days − 17.6% (95%CI=16.4-18.7).

**Table 2:** Characteristics of health risk behaviors of the sample (n=3992) according to sex.

	Female			Male		*p-value*
	n	%		n	%	
*Body mass index*						
Eutrophic	1254	83.7		2020	85.0	0.137
Overweight/obesity	245	16.3		356	15.0	

*MVPA*						
≥300min/week	447	29.0		451	18.4	<0.001
<300min/week	1097	71.0		1997	81.6	

*Sedentary behavior*						
Exposed	656	42.5		1209	49.4	<0.001
Not exposed	888	57.5		1239	50.6	

*F*+*V consumption*						
≥(2F+3V)	172	11.1		285	11.6	0.627
<(2F+3V)	1372	89.9		2163	88.4	

*Consumption of soft drinks*						
Once or more times per day	843	54.6		1453	59.4	0.002
Less than once per day	701	45.4		995	40.6	

*Consumption of cigarettes*						
Not exposed	1195	77.4		2096	85.6	<0.001
Exposed	349	22.6		352	14.4	

*Consumption of alcohol*						
Not exposed	794	51.4		1232	50.3	<0.001
Exposed	750	48.6		1216	49.7	

MVPA, moderate and vigorous physical activity; F, fruits; V, vegetables.

Table 3 shows the crude adjusted association between consumption of fruits and vegetables and other health risk behaviors in male students from Sergipe. Inadequate consumption of fruits and vegetables was associated to exposure to sedentary behavior, consumption of soft drinks and low level of physical activity. Adolescents who are exposure to sedentary behavior (OR=1.63; 95%CI=1.18-2.24), who consume soft drinks once or more times a day (OR=3.04; 95%CI=2.10-4.40) and who are insufficiently active (OR=1.98; 95%CI=1.43-2.73) were more likely to present low consumption of fruits and vegetables.

**Table 3:** Binary logistic regression to estimate the association of inadequate of fruits and vegetables consumption and other health risk behaviors among boys.

	Crude OR (95%CI)	*p-value*	Adjusted OR (95%CI)^a^	*p-value*
*Sedentary behavior*		0.004^b^		0.003^b^
Not exposed	1		1	
Exposed	1.61 (1.17-2.21)		1.63 (1.18-2.24)	
*Consumption of soft drinks*		<0.001^b^		<0.001^b^
Without consumption	1		1	
Consumption	3.07 (2.12-4.44)		3.04 (2.10-4.40)	
*MVPA*		<0.001^b^		<0.001^b^
≥300min/week	1		1	
<300min/week	1.97 (1.42-2.72)		1.98 (1.43-2.73)	
*Body mass index*		0.919		-
Eutrophic	1		-	
Overweight/Obesity	0.98 (0.63-1.51)		-	

OR, *Odds Ratio*; CI, confidence interval; MVPA, moderate and vigorous physical activity.

a Adjusted for age and skin color.

b *p*≤0.05, Teste de Wald.

Table 4 shows the crude adjusted association between consumption of fruits and vegetables and other health risk behaviors in female students from Sergipe. Inadequate consumption of fruits and vegetables was associated to consumption of soft drinks and overweight/obesity. Adolescents who consume soft drinks once or more times per day (OR=1.88, 95%CI=1.43-2.47) and who are overweight/obese (OR=1.63, 95%CI=1.19-2.23) were more likely to present low consumption of fruits and vegetables.

**Table 4:** Binary logistic regression to estimate the association of inadequate of fruits and vegetables consumption and other health risk behaviors among girls.

	Crude OR (95%CI)	*p-value*	Adjusted OR (95%CI)^a^	*p-value*
*Sedentary behavior*		0.777		-
Not exposed	1		-	
Exposed	1.04 (0.81-1.33)		-	
*Consumption of soft drinks*		<0.001^b^		<0.001^b^
Without consumption	1		1	
Consumption	1.88 (1.43-2.47)		1.88 (1.43-2.47)	
*MVPA*		0.089		0.113
≥300min/week	1		1	
<300min/week	1.30 (0.96-1.75)		1.28 (0.94-1.73)	
*Body mass index*		0.002^b^		0.002^b^
Eutrophic	1		1	
Overweight/Obesity	1.63 (1.19-2.22)		1.63(1.19-2.23)	

OR, *Odds Ratio*; CI, confidence interval; MVPA, moderate and vigorous physical activity.

a Adjusted for age and skin color.

b *p*≤0.05, Teste de Wald.

## Discussion

In this study, there was a high prevalence of inadequate consumption of fruits and vegetables among adolescents from Sergipe. Only 11.4% of young people met recommendations of daily consumption of these foods. This result is consistent with previous studies in other countries, which also revealed inadequate consumption of fruits and vegetables among adolescents. A study conducted in Europe^15^ revealed that adolescents consume half the recommended amount of fruits and vegetables. Likewise, research conducted in China^16^ verified that only 9% and 14% of adolescents met the minimum daily recommended values of vegetables and fruits, respectively.

Investigations on the consumption of fruits and vegetables in Brazil have shown that only 15% of students in the city of Florianópolis (SC),^17^ 6.5% in the city of Caruaru (PE),^18^ 5.3% in the city of Pelotas (RS)^19^ and 2.7% in the state of Santa Catarina^20^ consume the recommended amount of fruits and vegetables. In Pernambuco,^21^ one out of three adolescents does not consume or consumes fruits and vegetables at least once per day and 62.9% of them are daily exposed to excessive consumption of soft drinks. The National Adolescent School-based Health Survey (PeNSE)^22^ pointed out that 21% of students do not consume fruits and 26.8% do not eat vegetables in any day of the week.

Furthermore, there was a positive association between consumption of fruits and vegetables and variables related to other health risk behaviors. The results revealed that low levels of physical activity, exposure to sedentary behavior, consumption of soft drinks, and presence of overweight/obesity increase the likelihood of low consumption of fruits and vegetables, with some differences between boys and girls. Male adolescents were influenced by more factors (consumption of soft drinks, exposure to sedentary behavior and low level of physical activity) than females (consumption of soft drinks and overweight/obesity).

Inadequate consumption of fruits and vegetables was related to the habit of consuming soft drinks. Recent studies have shown that foods rich in sugars are inversely related to the consumption of fruits and vegetables.^23^^,^ 24 This finding corroborates results found in the last Brazilian Household Budget Survey - Brazilian POF,^25^ which indicated consumption of fruits and vegetables far below recommendations and high consumption of beverages with added sugar, such as juices and soft drinks.

The relationship between consumption of fruits and vegetables and exposure to sedentary behavior found in males students was similar to other studies such as that conducted with adolescents in Denmark,^26^ which verified association between unhealthy eating preferences and longer time spent watching television. In Austrália,^27^ the authors found significant association between TV time equal to or greater than two hours (≥2h/day) and consumption of soft drinks and salty snacks, and an inverse association between TV time (≤2h/day) and consumption of fruits and vegetables.

Low consumption of fruits and vegetables is related to low level of physical activity among male adolescents. Studies conducted with adults in Florianópolis (SC)^28^ also showed more adequate consumption of fruits and vegetables among those who practiced physical activity during leisure time compared to those inactive during leisure time. Similarly, a study conducted with adults in France^29^ revealed an inverse association between consumption of fruits and vegetables and unhealthy lifestyle behavior, especially low level of physical activity.

Low consumption of fruits and vegetables showed relationship with overweight and obesity in women. Similarly, in North-American^30^ adults, it was found that obesity was associated with inadequate consumption of fruits and vegetables. A study conducted with adults from Florianópolis (SC)^28^ showed that normal weight men were more likely to have adequate consumption of fruits and vegetables when compared to their overweight peers.

Promoting an increase in the consumption of fruits and vegetables among adolescents is a great challenge to public health, because it is associated with different behavioral factors, as well as with socioeconomic and demographic issues. However, it is necessary to be aware that consumption below recommendations can damage the growth and development potential of adolescents, as well as being a health risk factor. According to the guidelines designed to contribute to the prevention of diseases caused by nutritional deficiencies for the Brazilian population over two years of age,^6^ fruits and vegetables are a group of foods rich in vitamins, minerals and fibers, which should be in daily meals. Accordingly, increasing the production and supply of affordable and high-quality food to the population, ensuring the supply of fresh and nutritious foods in schools and including feeding and nutrition topics as a cross-sectional component in the elementary and high school curriculum are some strategies that can be developed by the government and by the food productive sector in order to promote health among adolescents.

This study presents some limitations which need to be considered such as: (a) the use of a questionnaire may increase the incidence of biases, (b) the generalization of data for all adolescents in Sergipe due to the fact that the study was restricted to school belonging to the state educational system and (c) the possibility of reverse causality due to the study design.

The findings of this study show that the prevalence of consumption of fruits and vegetables among adolescents from public schools of Sergipe is below current recommendations. From the perspective of public health, the factors associated with low consumption of these foods may subsidize health promotion programs in the school environment, also taking into account the differences between sexes.
